# Insights into Embryo Defenses of the Invasive Apple Snail *Pomacea canaliculata*: Egg Mass Ingestion Affects Rat Intestine Morphology and Growth

**DOI:** 10.1371/journal.pntd.0002961

**Published:** 2014-06-19

**Authors:** Marcos S. Dreon, Patricia E. Fernández, Eduardo J. Gimeno, Horacio Heras

**Affiliations:** 1 Instituto de Investigaciones Bioquímicas de La Plata (INIBIOLP), Universidad Nacional de La Plata (UNLP) – CONICET CCT, La Plata, Argentina; 2 Facultad de Ciencias Médicas, Universidad Nacional de La Plata, La Plata, Argentina; 3 Instituto de Patología B. Epstein, Cátedra de Patología General Veterinaria, Facultad de Ciencias Veterinarias, Universidad Nacional de La Plata, La Plata, Argentina; 4 Facultad de Ciencias Naturales y Museo, Universidad Nacional de La Plata, La Plata, Argentina; George Washington University School of Medicine and Health Sciences, United States of America

## Abstract

**Background:**

The spread of the invasive snail *Pomacea canaliculata* is expanding the rat lungworm disease beyond its native range. Their toxic eggs have virtually no predators and unusual defenses including a neurotoxic lectin and a proteinase inhibitor, presumably advertised by a warning coloration. We explored the effect of egg perivitellin fluid (PVF) ingestion on the rat small intestine morphology and physiology.

**Methodology/Principal Findings:**

Through a combination of biochemical, histochemical, histopathological, scanning electron microscopy, cell culture and feeding experiments, we analyzed intestinal morphology, growth rate, hemaglutinating activity, cytotoxicity and cell proliferation after oral administration of PVF to rats. PVF adversely affects small intestine metabolism and morphology and consequently the standard growth rate, presumably by lectin-like proteins, as suggested by PVF hemaglutinating activity and its cytotoxic effect on Caco-2 cell culture. Short-term effects of ingested PVF were studied in growing rats. PVF-supplemented diet induced the appearance of shorter and wider villi as well as fused villi. This was associated with changes in glycoconjugate expression, increased cell proliferation at crypt base, and hypertrophic mucosal growth. This resulted in a decreased absorptive surface after 3 days of treatment and a diminished rat growth rate that reverted to normal after the fourth day of treatment. Longer exposure to PVF induced a time-dependent lengthening of the small intestine while switching to a control diet restored intestine length and morphology after 4 days.

**Conclusions/Significance:**

Ingestion of PVF rapidly limits the ability of potential predators to absorb nutrients by inducing large, reversible changes in intestinal morphology and growth rate. The occurrence of toxins that affect intestinal morphology and absorption is a strategy against predation not recognized among animals before. Remarkably, this defense is rather similar to the toxic effect of plant antipredator strategies. This defense mechanism may explain the near absence of predators of apple snail eggs.

## Author Summary

Filled with nutritious substances to nourish the embryos, eggs of most animals are often the targets of pathogens and predators. An exception are the eggs of *Pomacea canaliculata* –known as the apple snail– which have hardly any predators. This freshwater snail is a serious aquatic crop pest in several continents, listed among the 100 worst invasive species. It is the host of a roundworm responsible for the rat lungworm disease causing human eosinophilic meningitis. The spread of this emerging infectious disease has been associated with the expansion of apple snails. They lay eggs above water level in bright pink-reddish masses, presumably a warning coloration. Indeed, eggs have chemical defenses, including neurotoxic and antinutritive proteins. The authors found that the ingestion of egg extracts adversely affects rat small intestine inducing large, reversible changes in the intestinal wall that limits the ability to absorb egg nutrients causing a diminished growth rate. Apple snail eggs are the first animal known to deter predators by this mechanism, but remarkably this defense is rather similar to the toxic effect of plant seeds proteins. These overlapping egg defenses that predators have not managed to overcome yet may partially explain the reproductive success of *P. canaliculata*.

## Introduction

The invasive apple snail *Pomacea canaliculata* (Lamarck, 1822) (Architaenioglossa, Ampullariidae) has become a serious aquatic crop pest in Asia and a vector of the rat lungworm *Angiostrongylus cantonensis* that causes human eosinophilic meningitis, a potentially fatal disease considered an emerging infectious disease. Unfortunately angiostrongyliasis (rat lungworm disease) continues to be reported in new regions beyond its native range which has been associated with the expansion of this snail [1]; [2]. *P. canaliculata* is the only freshwater snail listed among the 100 worst invasive species worldwide [3]. Their successful establishment in invaded areas may be related, among other factors, to their high fecundity, and the unusual characteristics of their eggs that increase the risk of the expansion of the disease. Females of *P. canaliculata* deposit hundreds of bright pink-reddish egg masses, each containing 30–300 eggs [4]; [5]. These egg clutches are remarkable in three respects: they are cemented outside the water, they are brightly colored and have virtually no predators, presumably because they have unusual defenses against predation [5]–[8]. Though filled with a perivitellin fluid (PVF) containing large amounts of carbohydrates and storage proteins (called perivitellins), these toxic eggs have no predators reported in their original South American range and only one in the newly colonized habitats in SE Asia: the fire ant *Solenopsis geminata* (Fabricius, 1804). The presence of these egg defenses [6]; [8]; [9] would explain the behavior of the snail kite *Rostrhamus sociabilis* (Vieillot, 1817) and Norway rat *Rattus norvegicus* (Berkenhout, 1769) that invariably discard the gland that synthesizes the egg defenses when predating on adult female *P. canaliculata* [10]–[14].

Work in the last two decades identified the perivitellins PcOvo and PcPV2 in the egg defenses against predation [6]; [8]; [13]; [15]–[17]. These are the most abundant perivitellins stored in large quantities in the PVF (57.0% and 7.5% of egg total protein for PcOvo and PcPV2, respectively) [15]. Both are resistant to proteolysis reaching the intestine in a biologically active conformation [6]; [8]. PcPV2 is a neurotoxic storage lectin with a strong lethal effect on selected neurons within the spinal cord of mice [9]; [18]. It is a novel combination of a tachylectin-like subunit with a membrane attack complex/perforin (MACPF)-like subunit, not reported in animals before [8]; PcOvo, on the other hand, is a storage carotenoprotein that provides the conspicuously reddish coloration of the clutches which presumably advertises to visual-hunting predators the presence of egg defenses (aposematic warning) [19]. In addition, PcOvo is a proteinase inhibitor limiting the ability of predators to digest egg nutrients. In fact, oral administration of purified PcOvo to rats significantly diminished rat growth rate presumably by a dual mechanism: the inhibition of trypsin activity (antidigestive role) and the resistance of the inhibitor to digestion by gut enzymes (antinutritive) [6]; [20]–[22]. A recent proteomic analysis of *P. canaliculata* PVF identified a small amount of over 50 other proteins, including two F-type lectins, many proteins involved in innate immunity in other mollusks and some with potential roles against insects and fungi [23].

As the epithelial cells along the digestive tract of animals are fully exposed to food contents, they are possible target sites for defense proteins. In this regard, plants have evolved a wide array of toxic dietary lectins that interact with the membrane glycoproteins of the luminal side of the gut of higher animals having an important role in plant defenses against predation [24]. There are, however, no reports in animals of such a defense mechanism [25].

With the aim to further understand the role of egg defenses of a host of the lungworm disease, in the present work we studied the effect of *P. canaliculata* PVF on the small intestine of rats. Through a combination of biochemical, histopathological, cell culture and feeding experiments, we provide evidence that oral administration of apple snail PVF adversely affects rat small intestine metabolism and morphology and consequently rat growth rate, presumably by proteins displaying lectin-like activity. This overall effect has not been found in other animals, but it is remarkably similar to that for plant seed lectins on the gastrointestinal tract of rats and other vertebrates.

## Materials and Methods

### Ethics Statement

All studies performed with animals were carried out in accordance with the Guide for the Care and Use of Laboratory Animals [26] and were approved by the “Comité Institucional de Cuidado y Uso de Animales de Experimentación” of the School of Medicine, UNLP (Assurance No. P08-01-2013).

### Eggs

Egg masses of *P. canaliculata* were collected either from females raised in our laboratory or taken from the wild in streams or ponds near La Plata city, Province of Buenos Aires, Argentina, between November and March of consecutive reproductive seasons. Only egg masses with embryos developed to no more than the morula stage were employed. Embryo development was checked microscopically in each egg mass as described elsewhere [16].

### Rats

All experiments with rats were performed using male Wistar rats from the Animal Facility of the School of Medicine of the National University of La Plata (UNLP), Argentina. Rats came from a colony started with the strain WKAHlHok (Hokkaido University, Japan). Six-week-old animals weighing 180±2 g at the start of the experiments were housed in cages with 12 h day-night cycle, temperature of 22±1°C and relative humidity of 45–60%.

### Preparation of PVF

Fertilized eggs were repeatedly rinsed with ice cold 20 mM Tris-HCl, pH 6.8, containing a protease inhibitor cocktail (Sigma Chemicals, St. Louis) and homogenised in a Potter type homogeniser (Thomas Sci., Swedesvoro, NJ). Ratio of buffer: sample was kept 5∶1 v/w. The crude homogenates were then sonicated for 15 sec and centrifuged sequentially at 10,000×g for 30 min and at 100,000×g for 60 min. The pellet was discarded and the supernatant comprising the egg PVF was equilibrated in 50 mM phosphate buffer pH 7.4 using a centrifugal filter device of 50 kDa molecular weight cut off (Millipore Corporation, MA) to eliminate potentially interfering compounds. Total protein concentration of the PVF (13.3 g/L) was measured by the method of Lowry *et al*. [27].

#### Isolation and purification of PcOvo

PcOvo was purified from the PVF of the eggs by high performance liquid chromatography (HPLC) (Hitachi Ltd., Tokyo, Japan). First, the sample was analyzed in a Mono Q HR 10/10 (Amersham-Pharmacia, Uppsala, Sweden) using a gradient 0 to1 M NaCl in a 20 mM Tris buffer. The PcOvo peak was then further purified by size exclusion chromatography (Superdex 200 HR 10/20, Amersham-Pharmacia, Uppsala, Sweden) using an isocratic gradient of sodium phosphate buffer 50 mM, 150 mM NaCl, pH 7.6. Purity of the single peak obtained was checked by native PAGE performed in a Mini-Protean III System (Bio Rad Laboratories, Inc.). Protein content was determined by the method of Lowry *et al*. [27].

### Experimental Protocols

#### Experiment 1

**Effect of egg PVF-supplemented diet on rat growth.** Groups of control and treated rats, 10 animals each, were fed *ad libitum* with a commercial diet for up to 7 days. Treated animals were administered PVF fraction (4 mg protein) in the water on a daily basis, while the control group received the equivalent volume of buffer. Food consumption as well as body weight were determined on a daily basis for each animal. The standard growth rate (SGR) was calculated as follows:
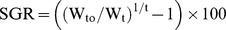
Where W_to_ is the initial weight, W_t_ is the final weight, and **t** is the time in days. [28].

#### Experiment 2

**Effect of egg PVF-supplemented diets on rat intestine size and morphology.** To establish the time-dependent changes induced by PVF on intestine, rats (groups of 5 rats each) were given either the commercial diet or the diet+PVF feed which contained 4 mg PVF protein rat^−1^ day^−1^ and sacrificed after, 4 or 10 days.

A similar setting was employed using PcOvo-supplemented diet at 4 mg PcOvo protein rat^−1^ day^−1^. Animals were sacrificed after 4 and 8 days and the absorptive surface of the intestine measured as described below (next section).

Rats were euthanized by CO_2_ inhalation in a closed chamber [26]. Care was taken to kill all animals at comparable times of the day (late morning). Intestines were removed and their length measured from pylorus to ileocaecal junction.

Sections of the first part of the duodenum were cut, washed several times with PBS to remove food and fixed in 4% formalin in phosphate buffered (pH = 7) for histological examination. In rats from day-4 treatment, sections for SEM analysis were also obtained (see scanning electron microscopy section).

To establish the time needed to recover after the changes induced by egg PVF on intestine, rats (groups of 5) were given either the commercial diet or a diet-and-egg PVF, which contained 4 mg protein rat^−1^ day^−1^. Animals were fed with the extracts for 10 days and then switched to a control diet and sacrificed 4 days after switching treated animals to control feed and processed as above.

### Histology and Morphological Measurements

Cylindrical tissue samples of the small intestine were post fixed in 10% neutral formaldehyde for 24 h at room temperature and then embedded in paraffin wax. Representative 5–7 µm sections were stained with haematoxylin and eosin for histological examination of general morphology. In addition, periodic acid Schiff (PAS) staining was performed to highlight carbohydrate distribution and goblet cells.

Fifty properly oriented villi and crypts from duodenum were selected at random from each animal and their length and width measured to calculate mucosal absorptive surface area following the method of Kisielinsky [29] whose results have no significant differences compared with the Harris method, widely used in rats [30]. The method considers a geometric mucosal unit of a cylindrical villous with rounded tip surrounded by cylindrical crypts. It assumes that the whole mucosa is an iteration of this unit, and the surface area can be calculated with mean values of structures that define the mucosal unit: villus length, villus width, and crypt width. Thus, the mucosal-to-serosal amplification ratio *M* was calculated considering these 3 variables, as follows:
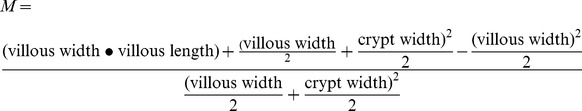



### Immunohistochemistry

Small intestine sections were assayed by immunohistochemistry (IHC) to evaluate cellular proliferation using a primary monoclonal mouse against the proliferating cellular nuclear antigen (PCNA) as a proliferation marker (Dako, Clon PC10). The antibody was diluted in 0.1% BSA in phosphate buffer and incubated overnight at 4°C. PCNA is a nuclear acid protein which functions as δ DNA polymerase helper. In the presence of PCNA and a replication C factor, δ DNA polymerase starts the synthesis of DNA and the progression of the cellular cycle.

Samples were incubated overnight at 4°C as mentioned above, and visualized using the LSAB kit (Dako Cytomation Lab, Carpinteria, USA) detection system which is based on a modified labeled avidin-biotin (LAB) technique in which a biotinylated secondary antibody forms a complex with peroxidase-conjugated streptavidin molecules. In short, after incubation with the appropriate primary antibody, a sequential 10 min incubation with an anti-mouse biotinylated antibody and peroxidase-labelled streptavidin is performed. Then staining is completed by incubation with 3,3′diaminobenzidine tetrahydrochloride (DAB) and H_2_O_2_. Positively stained cells showed a golden, dark-brown color. All sections were counterstained with Maeyer haematoxilyn before analysis. Primary antibody was replaced by normal mouse antiserum in control sections.

### Lectin Histochemistry

Small intestine sections were assayed with seven lectins (Table 1) (Lectin Biotinylated BK 1000 Kit, Vector Laboratories Inc., Carpinteria, CA, USA) namely: Con A (*Concanavalia ensiformis*), DBA (*Dolichos biflorus*), SBA (*Glycine max*), PNA (*Arachis hypogaea*), RCA-I (*Ricinus communis*-I), UEA-I (*Ulex europaeus*-I) and WGA (*Triticum vulgaris*) to reveal possible changes of the glycosylation pattern.

**Table 1:** Lectins used in this study and their major specificities.

Acronym	Lectin	Specificity	Concentration (µg/ml)
UEA-I	*Ulex europaeus-I*	α-L-Fuc	30
DBA	*Dolichos biflorus,*	α-D-GalNAc	30
PNA	*Arachis hypogaea*	β-D-Gal (β 1-3) D-GalNAc	10
SBA	*Glycine maximus*	α–D-GalNAc; β–D-GalNAc	30
WGA	*Triticum vulgaris,*	β-D-GlcNAc; NeuNAc	30
RCA-I	*Ricinus communis-I*	β-Gal	30
Con-A	*Concanavalina ensiformis*	α-D-Man; α-D-Glc	30

Specificities according to Goldstein and Hayes [50]. Fuc: Fucose; Gal: Galactose; GalNAc: N-Acetyl galactosamine: Glc: Glucose; GlcNAc: N-Acetyl glucosamine; Man: mannose; NeuNAc: Acetyl neuraminic acid (sialic acid).

In short, paraffin sections were deparaffinized with xylene dehydrated with 100% alcohol twice, 10 min each, and then endogenous peroxidase activity was quenched by incubating 5 min with hydrogen peroxide in methanol 0.3–3.0%.

They were then hydrated, washed in phosphate-buffered saline, and incubated with biotinylated lectins overnight. Then sections were washed with PBS, followed by 10-min incubation with streptavidin-HRP (streptavidin conjugated to horseradish peroxidase in PBS containing stabilizing protein and anti-microbial agents (Vector Laboratories Inc., USA). Finally the bound lectins were visualized by incubation during 4–10 min with a buffered Tris-HCl solution (0.05 M, pH = 6.0) containing 0.02% 3,3′-diamino-benzidine tetrahydrochloride (DAB) and 0.05% H_2_O_2_ (DAB; Dako, Carpinteria, USA). Positively-stained cells were demonstrated by a dark golden brown coloration. The sections were counterstained with Maeyer haematoxilyn.

### Scanning Electron Microscopy (SEM)

After 2-hour fixation in 2% (v/v) glutharaldehyde, samples were dehydrated in graded series of ethanol. Then ethanol was replaced by liquid carbon dioxide and samples were dried by critical point in a CP-30 (Balzers). Samples were gold metalized in a JEOL Fine Ion Sputter, JCF-1100. Observations and photomicrographs were obtained with a JEOL JSM 6360 LV SEM (Jeol Technics Ltd., Tokyo, Japan) at the Service of Electron Microscopy, Facultad de Ciencias Naturales y Museo, Universidad Nacional de La Plata, Argentina.

### Hemagglutinating Activity

Horse, goat, rabbit and rat erythrocytes were obtained from the animal facilities at the University of La Plata (UNLP). Blood samples were obtained by venous puncture and collected in sterile Elsever's solution (100 mM glucose, 20 mM NaCl, and 30 mM sodium citrate, pH 7.2) (Sigma Chemicals, St. Louis). Prior to use, erythrocytes were washed by centrifugation at 1500 g for 10 min in TBS buffer (20 mM Tris, 150 mM NaCl, pH 7.4). This procedure was repeated several times until the supernatant remained clear. Hemagglutinating activity was assayed in microtiter U plates (Greiner Bio One, Germany) by incubating a two-fold serial dilution of PVF (6 mg/mL) in TBS with 2% erythrocyte suspension in TBS at 37°C for 2 h. Results were expressed as the inverse of the last dilution showing visible hemagglutinating activity by naked eye.

### Cytotoxicity

Human colorectal adenocarcinoma cells (Caco-2) were cultured in Dulbecco's modified Eagle's medium (DMEM) (4.5 g/liter D-glucose) supplemented with 10% newborn calf serum, penicillin (10 U/mL), streptomycin (10 µg/mL), amino acids and vitamins (Life Technologies-Invitrogen). Cells were cultured at 37°C in a humidified atmosphere of 5% CO_2._ Culture medium was replaced every 2 days and subcultured by trypsinization when 95% confluent. Passages 60 through 65 were used for the experiments. Prior to each experiment, the viability of the cells was determined by trypan blue exclusion. Viability of every cell preparation exceeded 90% as determined by counting the stained cells.

The cytotoxic effect of the PVF on Caco-2 cells was evaluated using the 3-(4,5-dimethythiazol-2-yl)-2,5-diphenyl tetrazolium bromide (MTT) assay [31]. Cells were seeded in 200 µL of culture medium on 48-well plates at densities that ensured approximately 90% confluency after 24 h. Once cell cultures reached the desired confluence, 50 µl/well of a serial dilution of PVF (6 mg/mL) in PBS were added and incubated at 37°C for 24 h. Control wells were prepared with 50 µL/well of PBS. After treatments, culture medium was removed and cells were incubated with fresh medium containing 0.5 g/L of MTT at 37°C for 1 h. Plates were then centrifuged, the supernatant discarded and the cells were washed three times with PBS. Finally the cell monolayers were extracted with 200 µL/well of DMSO and the absorbance of each well recorded at 540 nm with background substraction at 640 nm in a microplate reader Multimode Detector DTX-880 (Beckman Coulter, Inc., CA, USA). Cell viability was expressed as control percentage [31].

%Viability  =  (OD treated cells/OD control cells) ×100

### Statistical Analysis

Data collected from all experiments were analyzed individually by either t test (histology) or ANOVA (bioassays) using Instat v.3.05 (Graphpad Software Inc.). Where significant differences between samples occurred, a post-hoc Tukey's HSD test was performed to identify the differing means. Results were considered significant at the 5% level.

### Accession Numbers

GenBank accession numbers for PcOvo subunits: JQ818215, JQ818216 and JQ818217; GenBank accession numbers for PcPV2 subunits: JX155861 and JX155862.

## Results

### Effect of Snail Egg Supplemented Diet on Rat Growth Rate

**Figure 1:**
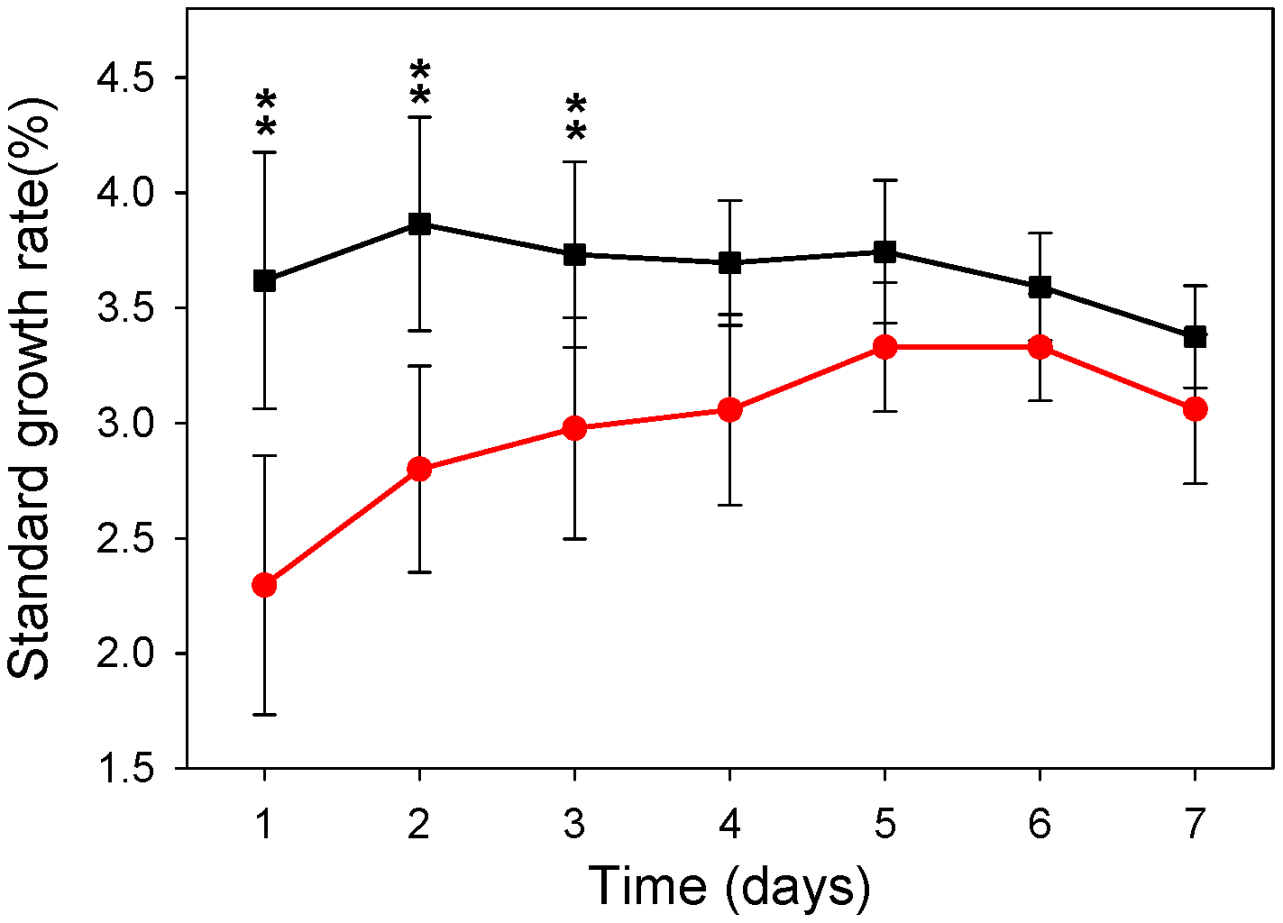
Effect of egg PVF-supplemented diets on Wistar rats' standard growth rate during the first 7 days. Control (black square), treated (red circle).Values represent the mean ±1 SD (n = 10); **p<0.01.

During the first 3 days of treatment with PVF, treated rats showed a significantly lower standard growth rate than the control ones (Fig. 1). This effect on growth rate disappeared after the fourth day of treatment and animals began to grow at the same rate as control groups. Daily food ingestion was similar in control and treated rats along the experimental period (results not shown).

### Effect of PVF on Rat Gastrointestinal Tract

**Figure 2:**
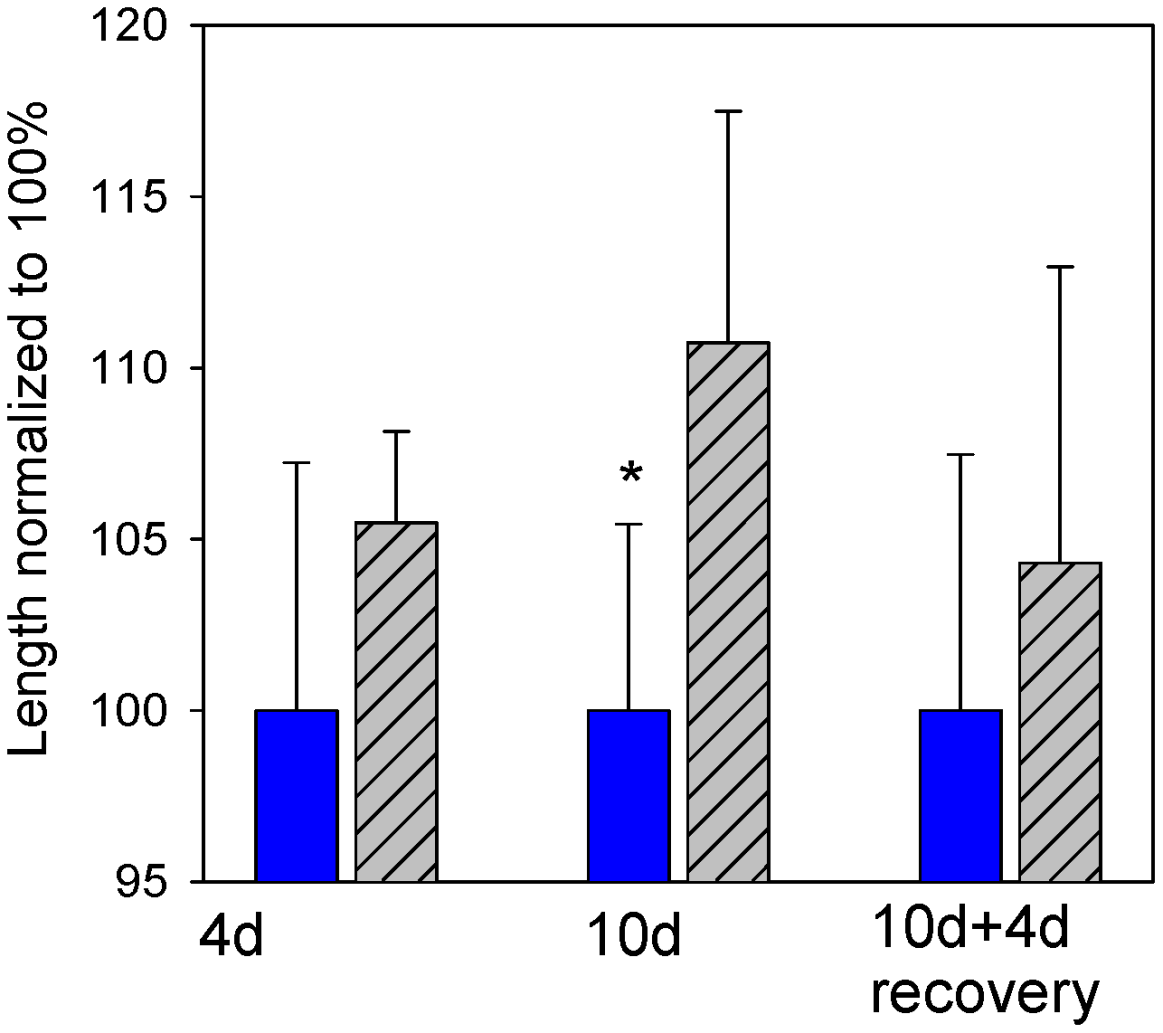
The effect of egg PVF on the length of the rat small intestine. Blue: Control; grey: PVF supplemented diet; 4 d: 4 day feeding; 10 d: 10-day feeding; 10 d+4d recovery: 10-day PVF feeding + 4-day diet switched to control conditions. *p<0.05. Bars represent the mean ±1 SD of 5 rats per group.

Oral administration of PVF for 10 days increased the mean intestinal length of the rats though a tendency was already evident after a 4-day treatment (Fig. 2). Within four days of switching the 10-day treated animals to a control diet, the total length of the small intestine returned to control values (Fig. 2). Crypt dimensions and general morphology of intestine were virtually restored to normal.

**Figure 3:**
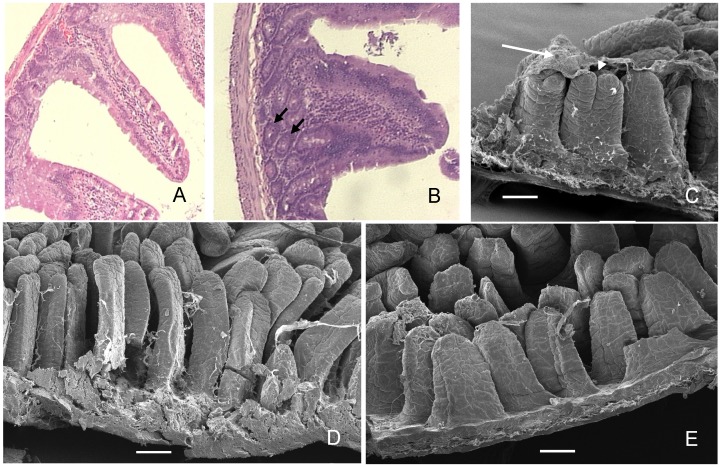
Changes in small intestine morphology after 4-day treatment with a diet supplemented with snail egg PVF. A,D control; B,C,E treated. Villi from treated animals are shorter and wider and displayed dome-shaped mucosal elevations which seem to connect two villi in a bridge-epithelial pattern (C, arrowhead) and are covered with more mucus (C, arrow). There is an increase in the proliferation of the basal epithelia in treated animals (B, arrows). A,B HE 10x; C,D,E SEM; bar  = 100 µm.

**Figure 4:**
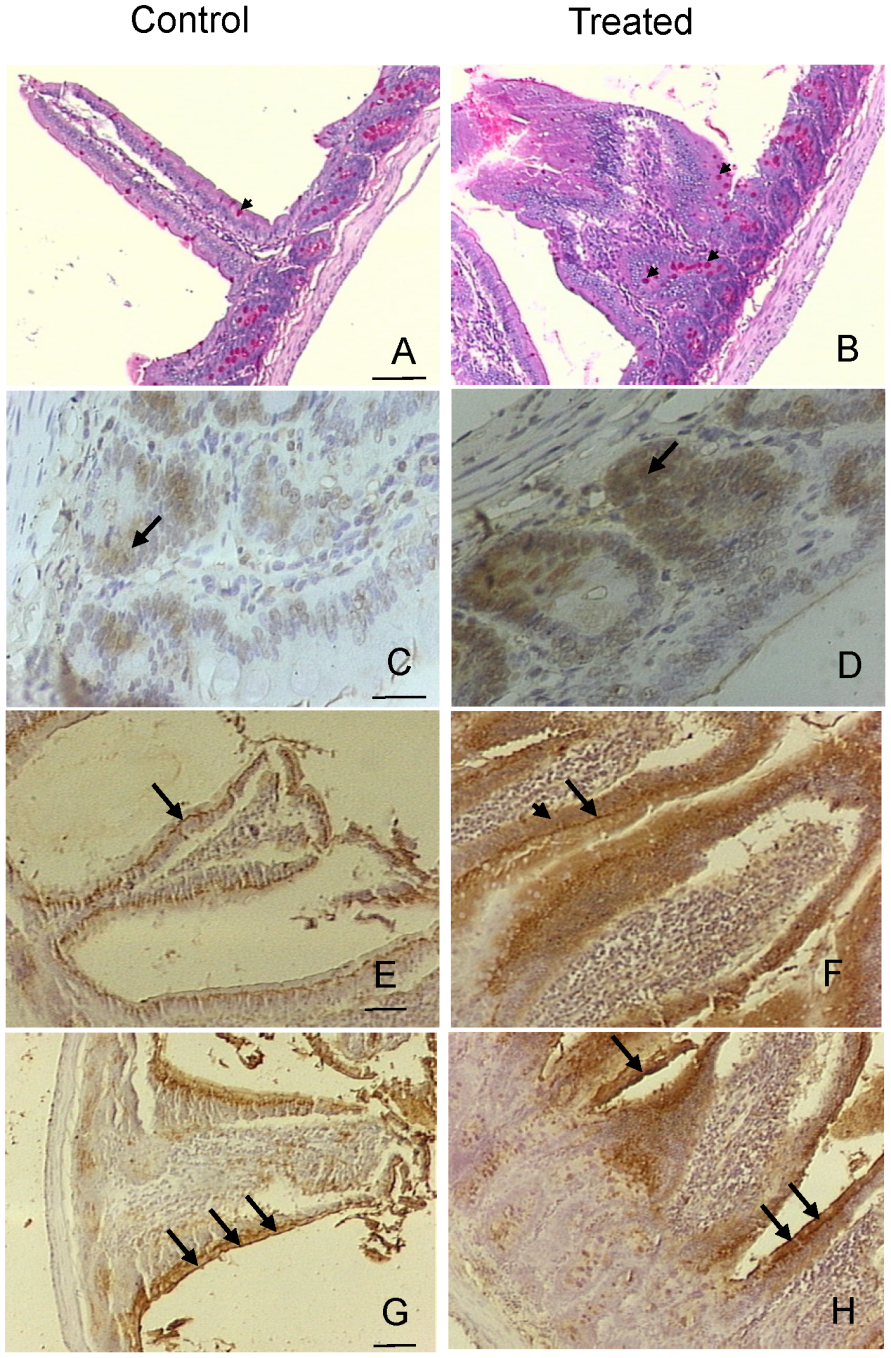
Effect of PVF administration on rat small intestine glucids, cell proliferation and glycosylation pattern. Rats were fed for 4 days on a diet without (A,C,E,G) or with (B,D,F,H) PVF containing 4 mg protein. A,B. PAS stain highlighting the goblet cells (arrowheads). C,D. PCNA Immunohistochemistry showing moderate and strong staining in control and treated samples, respectively. Arrows indicate the proliferation of the basal zone of the epithelium. E,F. PNA lectin histochemistry. Arrows: supranuclear zone, arrowhead: enterocyte; G,H. SBA lectin histochemistry in control and treated samples, respectively. Arrows indicate glycocalyx. The nuclei were counterstained with haematoxylin. A,B,E,F,G,H: Bar 100 µm; C, D: Bar 45 µm.

At day 4, samples from control animals showed the characteristic tall, finger-like villi, whereas villi from treated animals showed significantly less height and were wider with some proliferation in the basal zone of the epithelia. In certain areas of the epithelium of treated animals, altered villi with a double, fused or “tongue” shape, displaying a bridge pattern were observed by SEM and light microscopy (Fig. 3). PAS staining was moderate on the glycocalyx of villi and crypt enterocytes while the mucin of goblet cells showed a strong stain in both control and treated samples. Mucose epithelia from treated animals showed an increased number of goblet cells (Fig. 4 A,B).

### Scanning Electron Microscopy

SEM analysis of control and treated animals confirmed the remarkable differences on the length and width of the villi of treated animals (Fig. 3). The increased amount of mucus on the mucosa in the treated animals was also observed with this technique, as well as areas displaying conical, dome-shaped mucosal elevations which seem to connect two villi in a bridge-epithelial pattern (Fig. 3).

### Lectin and Immunohistochemistry

PCNA labeling showed moderate immunostaining in the basal areas of the epithelium from controls, while a stronger staining was evident on the treated animals. (Fig. 4 C,D arrows).

Intestinal epithelial cells were studied using a set of 7 lectins, of which PNA and SBA produced the most remarkable results (Fig. 4). PNA was strongly positive on the supranuclear region of enterocytes of control animals, while in treated animals its binding was observed not only in this region (strong staining) but also in the whole enterocyte (light staining) (Fig. 4, E,F arrows). Besides, SBA lectin binding was strong on the glycocalyx of the apical zone of the enterocytes of treated rats in comparison to the moderate staining in control group, indicating SBA-binding glycans were more expressed on enterocytes exposed to snail egg PVF (Fig. 4 G,H arrows).

### Absorptive Surface

When the effect of PVF on rat small intestine absorptive surface was quantified on histological sections, a significant decrease of the 4-day treated animals was observed while if the ingestion is continued for 8 days, the absorptive surface reverted to normal (Table 2). When rats were exposed to PcOvo, the small intestine did not show significant changes in absorptive surface for up to 8 days (Table 2) and villi morphology was normal.

**Table 2:** Small intestine mucosal absorptive surface (*M*) of rats ingesting control, PVF-supplemented diet or PcOvo-supplemented diets after 4 and 8 days.

	4 days	8 days
Control diet	7.13±1.28	7.38±1.78
PVF-supplemented diet	4.74±1.07*	7.19±2.49
PcOvo-supplemented diet	7.12±1.49	7.25±2.37

Values represent the mean ± SD of 5 rats (n = 50 cross-section/animal).

*p<0.05%; *M*: Mucosal-to-serosal amplification ratio.

### Hemagglutinating Activity

**Figure 5:**
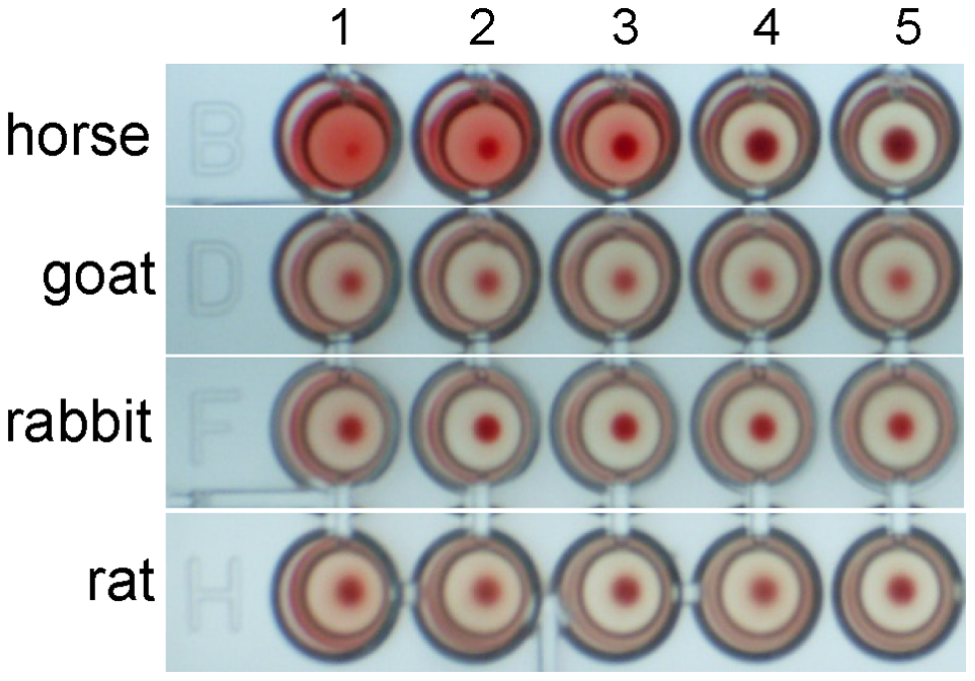
Hemagglutinating and hemolytic activity of *P. canaliculata* egg PVF against different types of mammalian erythrocytes. Well 1 to 5: 1.6, 0.8, 0.4, 0.2, and 0.1 µg/ml PVF protein.

The egg PVF of *P. canaliculata* showed hemagglutinating activity against horse red blood cells up to a protein dilution of 0.15 mg/mL, indicating the presence of active lectins. Moreover, a moderate agglutinating activity against rabbit and rat red blood cells was also observed at 0.6 mg/mL of PVF protein concentration (Fig. 5).

### Cytotoxic Effect of PVF on Caco-2 Cell

**Figure 6:**
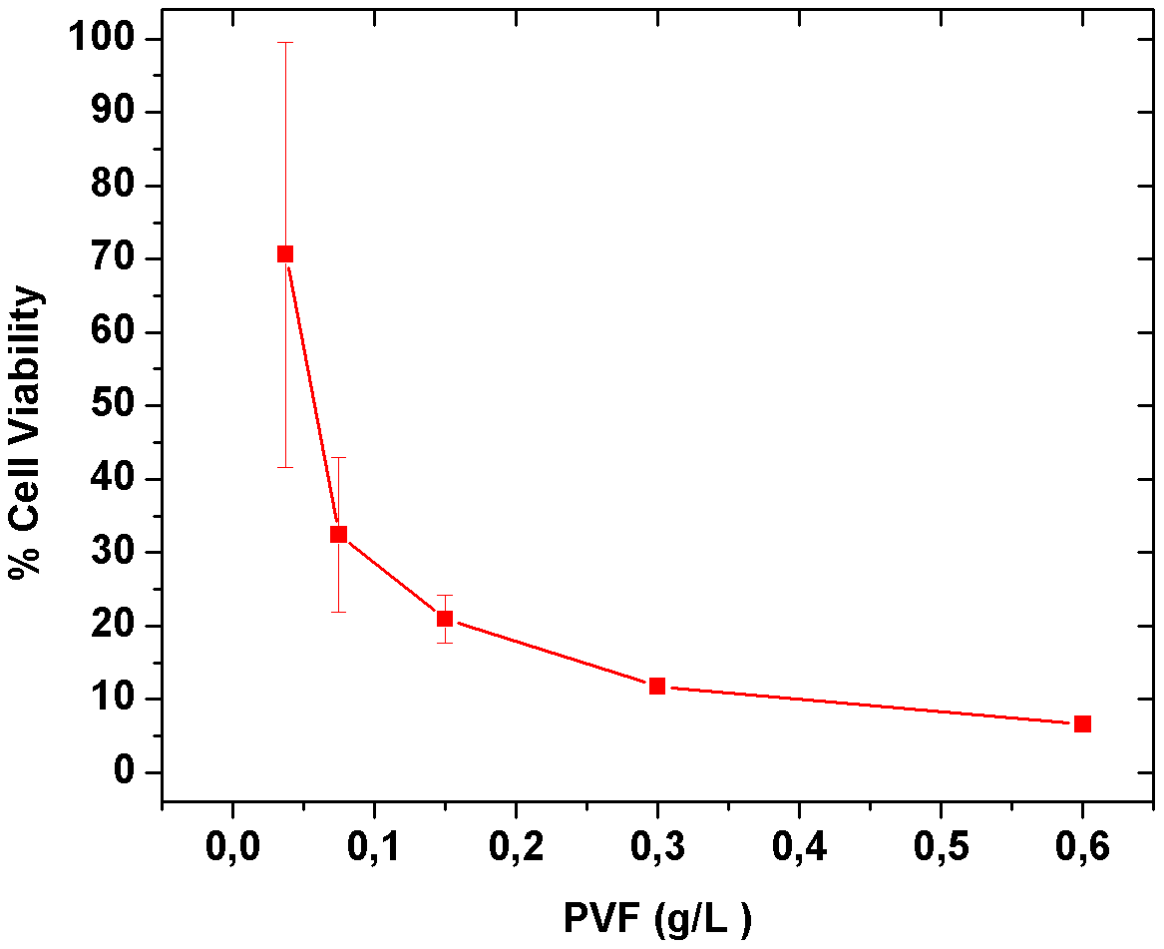
Cytotoxic effect of PVF on Caco-2 cells monolayers evaluated using MTT assay. Values are the mean ± SD (n = 6).

The MTT assay showed that PVF displays cytotoxic activity on Caco-2 cell monolayers in a dose-dependent manner. A very significant reduction of cell viability to only 6.6±0.6% in PVF-treated monolayers as compared to control ones was observed at a PVF protein concentration of 0.6 mg/mL (Fig. 6).

## Discussion

### The Effect of Apple Snail Egg PVF on Rat Intestine Resembles That of Dietary Plant Lectins

The ingestion of apple snail PVF severely affects the gastrointestinal tract rapidly causing a decrease in growth rate. Shortly after feeding a diet containing PVF the rat intestinal morphology undergoes a dramatic change. This included shorter and wider villi and fusion of villi by epithelial bridging, which might be related to the ability of the epithelial cells to stretch in order to cover denuded areas [32]. The observed enlargement of both villous and crypt thickness in treated animals was associated with the presence of hyperplasic crypts and hypertrophic mucosal growth changes. The notable increase in enterocyte proliferation and the presence of immature enterocytes in the crypts suggest increased mitotic activity in treated animals. This *in vivo* effect was further supported by the analysis of PVF cytotoxicity toward differentiated intestinal cells which indicates the presence of toxins somehow damaging these enterocytes. This damage in turn would induce the proliferative response observed at the crypt. Thus, the ingestion of PVF seems to interfere with gut and systemic metabolism, inducing hyperplasia and hypertrophy of the small intestine and alterations in organ function. Despite this effect on the gut being well established for plant toxic lectins [33] it has not yet been reported for the ingestion of animal proteins.

The enterocyte proliferation was also associated with changes of the glycosylation pattern revealed by the differential binding of the plant lectins PNA and SBA. PNA binds to the supranuclear portion of enterocytes where Golgi apparatus is located. It has been reported in humans orally intoxicated with PNA that the perturbation of cell kinetics and the more rapid cell migration and turnover of enterocytes may be reflected as synthesis of incomplete nascent glycoproteins, and expressed by altered PNA binding patterns [34]. This is also a well-known effect caused in rats intoxicated by other plant lectins that, as metabolic signals, can radically alter the pattern of glycosylation of the gut epithelium and thus further amplify their potent physiological effects [33]; [35]. These similarities between the effects of PVF and plant lectins lead us to look for lectin hemagglutinating activity in the PVF, which was found positive for some mammalian erythrocytes. This agrees with the recent identification of two putative lectins in a proteomic study of *P. canaliculata* PVF [23]. As mentioned before, one of these lectins, PcPV2, is the second most abundant egg protein. A functional study performed after its ingestion by rats showed that PcPV2 has the ability to withstand protease digestion, displaying structural stability within the pH range of the gastrointestinal tract of rats. Moreover, this toxic lectin binds to the glycocalyx of rat enterocytes *in vivo* and to Caco-2 cells in culture [8]. This interaction is also in agreement with the high cytotoxic effect of the snail PVF on Caco-2 cells observed in this study.

These properties are concurrent with those of many plant lectins which are resistant to mammalian gastrointestinal digestion and their toxicity is mainly attributed to the binding to the glycan surface of the small intestine epithelial cells, which leads to interferences with the digestion and anatomical abnormalities [35]–[38].

Besides lectins, PVF also contains the proteinase inhibitor PcOvo. When the effect of a PVF-containing diet on rat growth rate (Fig. 1) is compared with that of a PcOvo-containing diet [6], a larger decrease of rat growth rate was observed with PVF, indicating there are more defensive compounds acting synergistically. In addition, a PVF-supplemented diet, unlike a PcOvo-supplemented one, diminished intestinal absorptive surface. A literature survey reveals no information on animals in this regard but again, a reduction of the absorptive surface area was reported after the administration of diets containing plant lectins to rats, causing malabsorption of nutrients [33]; [39]. As a whole, the decrease on rat growth rate and changes in intestine morphology and absorptive surface caused by PVF ingestion together with the reported ability of PcPV2 toxic lectin to bind intestinal cells were rather similar to the effect observed on rodents fed with diets containing plant lectins strongly suggesting that PVF lectins may be involved in the observed effect of snail toxic eggs on the gut of the rat.

### Rats Gut Adapt to Prolonged Exposure to PVF

If the ingestion of PVF is continued, the rat growth rate becomes indistinguishable from that in control rats indicating an adaptation overcoming the antinutritional effect. Changes in the length of small intestine are often related with the difficulty in digesting the food. Greater length increases the transit time, thus maximizing digestion [40]. The adaptation to the PVF involved a time-dependent increase of the small intestine length, clearly observed after 10-day treatment. Similar effects were also observed in rats 3 days after administering diets containing phytohemagluttinin (PHA) from red kidney beans and other plant lectins [41]; [42]. However, those studies have shown that PHA-treatment of rats resulted in pancreas growth [42]; [43]. No such effect was observed in the current study (results not shown). In addition, the increase in mucous secretion suggests another adaptation allowing the isolation and protection of the intestinal surface from the toxic proteins. The change in length was virtually reverted 4 days after the elimination of the toxins from the diet along with the recovery of the normal tissue morphology. It is worth recalling that the mucosa of the small intestine is lined with epithelium that has the shortest turnover rate of any tissue in the body and in about 3 days' time the entire surface is covered with new cells [44]. Although there is no report of other animal lectins causing this effect, a fast remodeling of intestine by reversible effects on anatomy and morphology are known in rats and pigs administered diets containing plant lectins [41]; [45].

### Ecological Implications

Resting eggs are particularly vulnerable, since they are most attractive to potential parasites and predators and may lack an active defense system (because of their inactive metabolic state). Apple snails seem to have evolved passive defense systems to protect their developing embryos; the preferential accumulation of large quantities of lectins, and protease inhibitors is certainly indicative of that strategy. Moreover, it is believed that the main antinutrients responsible for reducing the nutritional value of many plant seeds are a combination of lectin and trypsin inhibitors [46]. Similarly, in apple snail eggs these two types of proteins may be also the main factors responsible for this effect. This further highlights the previously reported similarities between apple snail egg and plant seed embryo defenses [6]; [8]. In a broader view, the overall effect of *P. canaliculata* PVF on rats bears many similarities with the effect of plant dietary lectins not only against mammals but also birds, insects and nematodes, preventing these predators from digesting and incorporating nutrients from the tissues consumed [47]–[49]. Unlike plants, *P. canaliculata* advertises its defenses by a conspicuous coloration of the egg masses. Eggs indeed seem to have a large number of defensive proteins against predation, such as other protease inhibitors, chitinases, glycanases, lectins and antifungal proteins, as the analysis of the PVF proteome revealed [23]. Interestingly, all of these defensive proteins are also present in many plant seeds. It is possible that the combined effect of these defensive perivitellins -some targeting the digestive system while others aiming at other organs- may be an evolutionary adaptation. Although these defenses may not completely protect an egg from consumption, they may very well confer an advantage that increases its fitness helping to explain the virtual absence of egg predators. With more than 80,000 species, gastropods are the second largest class of animals after insects. It is therefore not surprising that a better understanding of gastropod egg biochemical defenses, little studied to date, is unveiling novel strategies not previously recognized among animals. In this regard, this study provides insights on the unique defenses against predators of a snail egg that are advertised by conspicuous coloration, and suggests that the acquisition of this protection may have conferred a survival advantage. This places apple snail eggs in the “winning side” of the predator-prey arms race.

### Conclusions

In this work we demonstrate that the oral administration of apple snail egg PVF promotes alterations in rat growth rate and small intestine morphophysiology for short periods, whereas prolonged exposure to the toxic PVF induces an adaptation overcoming the antinutritonal effects. This defense has not been reported in animals before, but resembles those well established for plant seeds.

The severe effects of PVF on digestive tract adds another line of defense to the previously reported suite of biochemical defenses of apple snail eggs. This study helps to explain the near absence of predators and their successful establishment in invaded areas.
